# Therapy free of cells vs human mesenchymal stem cells from umbilical cord stroma to treat the inflammation in OA

**DOI:** 10.1007/s00018-022-04580-z

**Published:** 2022-10-20

**Authors:** Miriam Morente-López, Rocío Mato-Basalo, Sergio Lucio-Gallego, Lucía Silva-Fernández, Alba González-Rodríguez, Fco. Javier De Toro, Juan A. Fafián-Labora, María C. Arufe

**Affiliations:** Grupo de Terapia Celular y Medicina Regenerativa, Dpto. de Fisioterapia, Medicina y Ciencias Biomédicas, Facultad de Ciencias de La Salud, Universidade da Coruña, INIBIC-CHUAC, CICA, 15006 A Coruña, Spain

**Keywords:** Mesenchymal stem cells (MSC), Extracellular vesicles (EV), miR-21-5p (miR-21), Syndecan-1 (SDC1)

## Abstract

Osteoarthritis (OA) is closely linked to the increase in the number of senescent cells in joint tissues, and the senescence-associated secretory phenotype (SASP) is implicated in cartilage degradation. In the last decade, extracellular vesicles (EV) in combination with the use of miRNAs to modify post-transcriptional expressions of multiple genes have shown their utility in new therapies to treat inflammatory diseases. This work delves into the anti-inflammatory effect of extracellular vesicles derived from mesenchymal stem cells (MSC) previously modified to inhibit the expression of miR-21. We compare the efficacy of two treatments, MSC with their miR-21 inhibited through lentiviral transfection and their EV, against inflammation in a new OA animal model. The modified MSC and their EV were intraperitoneally injected in an OA animal model twice. One month after treatment, we checked which therapy was the most effective to reduce inflammation compared with animals untreated. Treated OA model sera were analyzed for cytokines and chemokines. Subsequently, different organs were analyzed to validate the results obtained. EV were the most effective treatment to reduce chemokines and cytokines in serum of OA animals as well as SASP, in their organs checked by proteomic and genomic techniques, compared with MSC alone in a statistically significant way. In conclusion, MSC-miR-21^−^-derived EV showed a higher therapeutic potential in comparison with MSCs-miR-21-. They ameliorate the systemic inflammation through inactivation of ERK1/2 pathway in OA in vivo model.

**Graphical abstract:**

Workflow of the realization of the animal model of OA by injecting cells into the joint cavity of the left knee of the animals, which produces an increase in serum cytokines and chemokines in the animals in addition to the increase in SASP and markers of inflammation. Inhibition of miR-21 in MSCs, from the stroma of the human umbilical cord, by lentivirus and extraction of their EVs by ultracentrifugation. Finally, application of MSC therapy with its miR-21 inhibited or its EVs produces a decrease in serum cytokines and chemokines in the treated animals, in addition to an increase in SASP and markers of inflammation. The cell-free therapy being the one that produces a greater decrease in the parameters studied

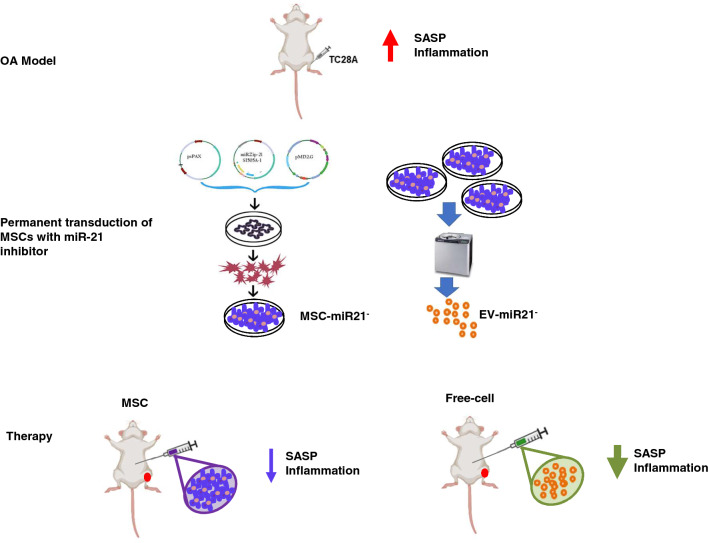

## Introduction

Mesenchymal stem cells (MSC) have proven immunomodulatory properties, and in the last decade, their ability to release extracellular vesicles (EV) has been widely studied. EV libered from MSC would generate a suitable microenvironment to enhance their anti-inflammatory capacity. EV, which include small extracellular vesicles, contain multiple bioactive molecules such as miRNA. This content is the reason why EV act as a vehicle for cell–cell communication to influence cellular activities, regulating the expression of target genes in the receptor cells [1].

Our group has reported that miR-21-5p (miR-21) is abundant in extracellular vesicles from MSC of the aging rats. We demonstrated the link between miR-21 expression contained in EV and damage-associated molecular patterns by aging (DAMPs), after conducting functional experiments inhibiting its expression. These results indicated for the first time that MSC-derived EV have significant age-dependent differences in their immune profiles and the miR21 has been shown to play a key role in the regulation of immune and inflammatory responses [2].

Osteoarthritis (OA) is the most common joint disease and a degenerative disease. The accumulation of senescent cells in the cartilage is implicated in the development of this pathology [3]. The senescent cells have a senescence-associated secretory phenotype (SASP) implicated in the intercellular communication between cells [4]. It was determined that the systemic inflammation is increased in in vivo OA models [5, 6].

SASP is being seen to offer a new perspective as a marker for the progression of many age-related diseases. Being able to give us an idea of the state of inflammation and complexity of multiple diseases associated with age from cancer to neurodegeneration [7], OA included [8, 9]. In this work we studied the role of miR-21 in the inflammatory profile and SASP in a rat OA model. In addition, we compared the efficacy of two treatment, MSC with their miR-21 inhibited through lentiviral transfection and their derived EV, against inflammation in a new OA animal model and discern the mechanism could be helping to reduce inflammation in our OA model when we used them as cell-free therapy. It is of particular interest to understand the basic biology of inflammation modulation by studying the nature of miR-21 in EV derived from MSC. Understanding all of these properties of extracellular vesicles makes them especially attractive for the development of new therapeutic approaches.

## Material and methods

### Tissue collection

Human umbilical cord stromas were obtained from cesarean surgeries performed on healthy women, these tissues were obtained withfully informed consent and ethical approval by the supervision from the Clinical Experimentation Ethics Committee (CEEC # 2021/010). All surgeries were performed in the Maternity Facility at Complejo Hospitalario Universitario A Coruña.

### Isolation and characterization of MSC

MSCs were isolated from human umbilical stromal tissue using a protocol developed by our group [10]. Briefly, the isolation was performed through explants after three short incubations with a cocktail containing 1.2 U/mL dispase and 112 U/mL type I collagenase (all from Sigma–Aldrich). After 3 days, the explants were removed from the plate, leaving attached the MSC, which are cultured in monolayer in Dulbecco's modified Eagle's medium (DMEM) with 10% FBS, 1% penicillin, and 1% streptomycin. The cells were used in the experiments when they reached 90% confluence.

The characterization of MSC was performed by biological differentiation towards three cell lines FACS analysis of membrane MSC markers and by PCR to check pluripotency markers genes (Table 1).

**Table 1:** Specific primers for real-time reverse transcriptase polymerase chain reaction (RT-PCR) amplification

Gene name	Fw primer	Rv primer
*Nanog*	tctccaacatcctgaacctca	ttgctattcttcggccagtt
*Sox9*	gtacccgcacttgcacaac	tgcctctcgttcagaagtctc
*Oct4*	gaaacccacactgcagatca	cggttacagaaccacactcg
*IL-6*	ccggagaggagacttcagag	cagaattgccattgcacaac
*HMGB1*	ccggatgcttctgtcaactt	ttgatttttgggcggtactc
*S100A6*	tgatccagaaggagctcacc	agatcatccatcagccttgc
*IL-1β*	tacctgtcctgcgtgttgaa	tctttgggtaatttttgggatct
*S100A2*	ccacaagtacgccagtcaag	agaatccaccagaaccaggg
*S100A3*	ttgccagcctctgtctctac	cagagagagtgggaagggtg
*MMP9*	ggcgctcatgtaccctatgt	gccattcacgtcgtccttat
*HPRT*	agcagtacagccccaaaatg	ggcctgtatccaacacttcg

### Biological differentiation

Chondrogenic differentiation was performed using MSC at P1 which were seeded into 6-well plates (Sarstedt) at 2 × 10^5^ cells per well in DMEM with 15% knockout serum (Gibco, Invitrogen), 5 mg/mL ascorbic acid, 6 μg/mL transferrin, 10 μM dexamethasone, 1 × 10^−7^ M retinoic acid (all form Sigma–Aldrich), and 1 ng/mL recombinant human transforming growth factor-β3 (TGF-β3) (ProSpec-Tany TechnoGene; Deltaclon). Adipogenic and osteogenic differentiation were performed with MSCs at P1 seeded in 6-well plates (Sarstedt) at 2 × 10^5^ cells per well in adipogenic or osteogenic commercial medium (Cambrex, Lonza), following the manufacturer's instructions, to assess the mesodermal differentiation potential. Control MSC were grown under DMEM with 10% knockout serum (Gibco, Invitrogen), 1% penicillin, and 1% streptomycin (Sigma–Aldrich). All the mediums were changed every 3 days.

### Immunohistochemistry analysis

MSC following the different differentiations were stained with Hematoxylin–Eosin, Red Alyzarin and Oil Red O after 21 days in culture (all from Sigma–Aldrich). All cultures were fixed in 10 mM sodium periodate, 2% paraformaldehyde, 75 mM l-lysine dihydrochloride, and 37.5 mM dibasic sodium phosphate (all from Sigma–Aldrich) at pH 7.4 for 15 min at room temperature, then air dried. The differentiated cells were stained with a filtered solution of 0.3% Oil Red O to reveal lipid droplets or with Alizarin Red S 2% aqueous solution at pH 4.2 (Sigma–Aldrich) for 3 min to assess calcium deposits or Safranin O to check proteoglycan formation in the cells.

Sections of 5 μm of knee joint were cut in the microtome after inclusion in paraffine. The histological sections from the in vivo model knee joint were immersed in a tray with Weigert's iron hematoxylin solution, then passed through acid alcohol and washed with water. Continue the colouring process by immersing the sheet in fast green, it is washed with acetic acid and now it is immersed in Safranin O. To finish the process, it is dehydrated using alcohols at different concentrations in ascending order. The last step required xylene for the sample to clarify.

The slides are conditioned with Canada balsam or similar to be observed under the microscope (Olympus). With this technique, the nuclei are stained black, the bone green and the cartilage where the proteoglycans are found red.

### FACS analysis

The cells were washed twice with PBS and incubated for 1 h at RT with the following direct antibodies: phycoerythrin (PE) mouse anti-human CD34 (1:20; DakoCytomation); FITC mouse anti-human CD45 (1:20; BD Pharmingen); FITC mouse anti-human CD105 (1:100; Serotec); PE-Cy5.5-conjugated mouse anti-human CD90 (1:20; BD Pharmingen); PE-conjugated anti-human CD73 (1:20; BD Pharmingen). The stained cells were then washed twice with PBS and 10 × 10^5^ cells were analyzed with a FACSAria flow cytometer (BD Bioscience). FACS data were generated by DIVA software (BD Bioscience). Negative control staining was performed using FITC-conjugated mouse IgG1k isotype, PE-conjugated mouse IgG1k isotype, and PE-Cy5.5-conjugated mouse IgG1k isotype (all from BD Pharmingen).

### Isolation and characterization of MSC-derived EV

Three days before starting the ultracentrifugation process of EV extraction from MSC in culture, the regular fetal bovine serum was replaced by exosome-depleted fetal bovine serum to guarantee that the EV did not originate from the serum. The supernatant is collected and centrifuged at 3000×*g* for 10 min in a Beckman Coulter Allegra X-22 Centrifuge. The supernatants are then filtered through a 0.22-µm filter and added to new ultracentrifuges tubes. Two serial ultracentrifuges with Hitachi CP100NX are performed, at 100,000×*g* for 2 h at 4 °C, the last pellet is re-dissolved in 200 µL of PBS to posterior analysis.

### EV measurement

Size distribution and concentration measurement of EV were conducted on a second generation nanoparticle tracking analysis (NTA) instrument, the ZetaView (Particle Metrix, Germany) with a 488-nm laser and software ZetaView 8.04.02. Temperature was controlled at 24 °C. All parameters were recommended by the manufacturer for EV analysis. After initial wash and calibration, samples were resuspended in 200 µL of PBS.

### Transmission electron microscopy

Purified EVs were covered with Formvar-carbon-coated EM grids to promote the absorption of EVs onto membranes over 20 min in a dry environment at room temperature. The grids were then placed directly on a drop of 1% glutaraldehyde and incubated for 5 min to remove the negative background. The grids were washed seven times with distilled water for 2 min each and examined using a JEOL JEM 2010HR with a built-in semiSTEM unit, CCD camera 780-AJ08HA from Gatan.

### Permanent transduction of MSCs with miR-21 inhibitor

The Lenti-X™ Lentiviral Expression System (Clontech Laboratories Inc.) was used following the manufacturer's protocol. One day before transfection 4 × 10^6^ HEK293T producer cells were placed on 100 mm plates in penicillin/streptomycin-free DMEM supplemented with 5% FBS. The following day, three different Polyethyleneimine (PEI) based transfections were performed using HEK293T cells to produce the retrovirus carrying either miR-21 inhibitor or mimic. Viral production was achieved by the assembly of a retroviral packaging system that contains the packaging plasmid pUMVC (Addgene #8449), the envelope plasmid pCMV-VSV-G (Addgene #8454), and the target plasmid SBI´s pGreenPure (#S1505A-1, SBI System Science) with the miR-ZIP hairpin cloned #mZIP21-PA-1 to inhibit miR-21 or #mZIP000-PA-1 as a control. Viral supernatants were collected after 48, 72 and 96 h of transfection, and were added on MSC in the presence of polybrene at a concentration of 8 μg/ml. The infection medium was replaced with regular DMEM after 4 h of infection. Drug selection began at 48 h after the transduction, and the selection process continued for 10 days until 95% of cells were GFP positive. The cells were incubated overnight with the transfection mixture, then washed with PBS and incubated with 8 mL of fresh complete growth medium. Viral supernatants were collected at 48 h, 60 h and 72 h following transfection, centrifuged, filtered to remove cell debris and stored at 4 °C until transduction.

MSC were plated in 100 mm plates at 6 × 10^6^ cells per plate. After 1 day, the cells were 70% confluent. The cells were incubated sequentially with the 48 h, 60 h, and 72 h viral supernatants for 12 h. Following the last transduction, the cells were washed and incubated with fresh growth medium to allow puromycin-resistance expression. Two days later, puromycin selection was performed by incubating the cells in growth media supplemented with 1 μg/mL puromycin (Clontech Laboratories Inc.) for 5 days. After selection, transduced cells were washed and allowed to recover in complete media for 2 days and they were called MSC-miR-21^−^.

### In vivo model of OA

12 two-month-old animals were injected with 1 × 10^5^ human chondrocytes cell line (TC28a2) (from Sigma–Aldrich, Madrid, SP) in 50 µL of PBS in their intra-articular capsule in their left knee to disrupt the joint and create an OA model. After 1 month from the chondrocyte injection the animals were splitted in three random groups of 4 animals. One group was ip injected with PBS to create the PBS group; other group was injected with MSC-miR-21^−^ and another group was ip injected with MSC-miR-21^−^-derived EV. The ip injections were repeat 1 week later form the first one. All animals were euthanized 1 month after their last ip injections. The euthanize was done by inhaling CO_2_ in a suitable chamber for it. All animal procedures and handling were supervised by veterinarians of the Animal Experimentation Unit of CHUAC and the approval from Animal Experimentation Ethical Committee has been done (AEEC#2020/R1). Serum from the OA animal model was collected after euthanizing, centrifuged at 12,000×*g* for 10 min, transferred into polypropylene tubes and stored at − 80 °C.

### Luminex immunoassay

Cytokine & Chemokine 22-Plex Rat ProcartaPlex™ Panel (#EPX220-30122-901, Invitrogen, London, UK) was measured in serum from OA animal model (Control (four control animals without any treatment), PBS (four control OA animals treated with PBS), MSC-miR-21^−^ (four OA animals treated with MSC-miR-21^−^) and EV-miR-21^−^ (four OA animals treated with MSC-miR-21-derived EV)), following the manufacturer’s instructions. Luminex multi-factor detection by xMAP technology following the manufacturer's instructions was performed at 1 µL of each sample.

### Reverse transcription quantitative PCR analysis

Total RNA from culture cells was isolated with TRIzol^®^ reagent (Thermo Fisher Scientific, Waltham, MA, USA). For miRNA detection, cDNA was generated from DNaseI-treated RNA, using a QuantiMir RT Kit (System Biosciences, Palo Alto, CA, USA), according to the manufacturer’s instructions. PCR products were amplified using specific primers for miRNAs, miR-21-5p (rno481342_mir) and U6 (Rn01526055_g1) small nuclear RNA (Thermo Fisher Scientific, Waltham, MA, USA). The amplification programme consisted of an initial denaturation at 50 °C for 2 min, followed by 95 °C for 10 min, and 50 cycles of annealing at 95 °C for 15 s and extension at 60 °C for 1 min. Primers for the amplification of genes are described in Table 1. The amplification programme consisted of an initial denaturation at 92 °C for 2 min, followed by 40 cycles of annealing at 95 °C for 15 s; annealing at 55–62 °C, depending on the gene, for 30 s; and extension at 72 °C for 15 s. PCRs were done in triplicate, with each set of assays repeated three times. To minimise the effects of unequal quantities of starting RNA and to eliminate potential sources of inconsistency, relative expression levels of each gene were normalised to ribosomal protein (HPTR) or U6 small nuclear RNA using the 2^−ΔΔCT^ method [11]. Negative control used with water free-RNAses instead of cDNA.

### Immunofluorescence analysis

Liver tissues from each animal were frozen in OCT embedding matrix (BDH Chemicals, Poole, UK) until their posterior analysis. Full-depth sections (thickness 4 μm) were cut with cryostat. The sections were immunostained with antibody SDC1 (1:100 Rabbit mAb #12922 from Cell Signaling Tech. Madrid, SP) after that were washed with PBS, then incubated with Anti-rabbit IgG (H + L), F(ab')2 Fragment (1:1000 #4414 Alexa Fluor^®^ 647 Conjugate from Cell Signaling Tech. Madrid, SP) for 30 min at room temperature. The pictures shown were done by an Olympus microscopy.

### Immunoblot analysis

Immunoblot analysis was performed with 40 μg total protein extracted from MSCs or 20 μg total protein extracted from MSC-derived EV or 40 μg total protein extracted from liver and spleen. Proteins were separated according to their molecular weight using sodium dodecyl sulphate–polyacrylamide gel electrophoresis (SDS-PAGE), with the percentage bis-acrylamide (Sigma–Aldrich, St. Louis, USA) of the resolving gels being determined by the size of the proteins. Proteins were then transferred to nitrocellulose membranes using a semi-dry method, using buffer with 20% (v/v) methanol (Panreac, Barcelona, Spain) for small proteins (< 100 kDa) or 10% (v/v) methanol (Panreac, Barcelona, Spain) for large proteins (> 100 kDa). Nitrocellulose membranes were then incubated for 1 h with agitation at room temperature in blocking buffer, consisting of 5% (w/v) bovine serum albumin (BSA) for phospho-proteins and 5% (w/v) milk (Sigma–Aldrich, St. Louis, USA). The membranes were probed with antibodies diluted in blocking buffer at 4 °C overnight. The following day, the membranes were washed three times for 5 min with Tris-buffered saline with 0.1% (v/v) Tween^®^ 20 (TBST). The membranes were then incubated for 1 h at room temperature in horseradish peroxidase (HRP)-conjugated secondary antibodies diluted in blocking buffer. Next, the membranes were washed three times in TBST buffer for 5 min with agitation and twice using Tris-buffered saline (TBS) for 5 min with agitation. An Amersham ECL Western Blotting Analysis System (GE Healthcare, Little Chalfont, UK) was used to visualise protein-binding antibodies. The blots were probed with antibodies directed against ERK1/2, phosphor-ERK1/2, AKT, phosphor-AKT, and SDC1 (Cell Signaling Technology, Beverly, MA, USA); Tsg101, calnexin and CD63 (Abcam, Cambridge, MA, USA); and β-actin (Sigma–Aldrich, St. Louis, MO, USA). Adequate concentrations for each antibody were determined empirically. Blot images were digitised using a LAS 3000 image analyser (GE Healthcare, Little Chalfont, UK). Densitometry analysis of band intensities was performed using ImageQuant 5.2 software (GE Healthcare, Little Chalfont, UK).

### Enzyme-linked immunosorbent assay (ELISA)

ELISA kits specific for SDC1 (DuoSet^®^ cat N°DY206-05, R&D Systems, Minneapolis, MN, USA) were used following the manufacturer’s instruction. 100 µL of serum from an OA animal model treated with MSC-miR-21^−^ or their derived EV or without treatment were measured. A NanoQuant microplate reader (Tecan Trading AG, Switzerland) was used.

### Statistical treatment of the data

Data analyses and mapping were conducted via GraphPad Prism 8.0 statistical software (GraphPad Software Inc., San Diego, CA, USA). The results are expressed as the mean ± standard deviation. We will compare the means of the variables studied between the different treatments after checking normality with the Kolmogorov–Smirnov test, with the *T* test for paired samples or the Wilcoxon test, according to procedure. 2way ANOVA was performed between treatments in vivo OA model. A value of *P* < 0.05 was indicative of a significant difference.

## Results

### Characterization of MSC from umbilical cord stroma and their EV

**Fig. 1:**
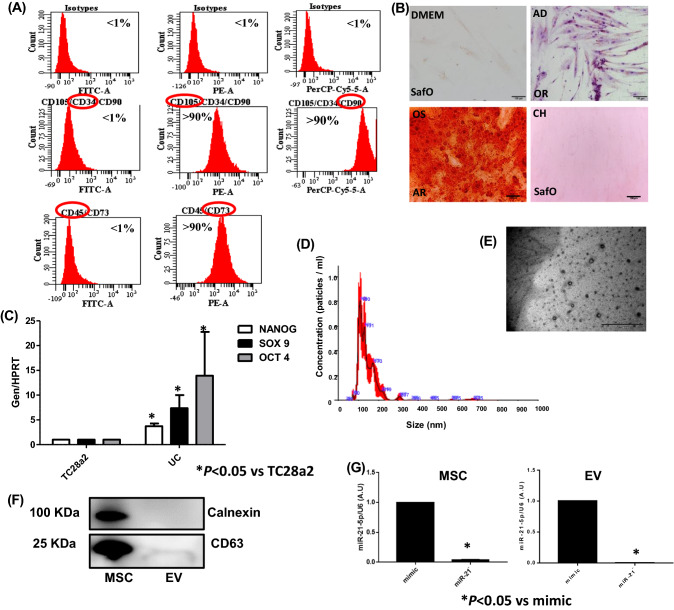
Characterisation of MSCs and their derived EV from human umbilical cord estroma. **A** One representative fluorescence-activated cell sorting (FACS) assay is shown. The antibody is indicated at the top of each plot and its linked fluorochrome at the bottom. Positive MSC markers (CD105, CD90 and CD73) and negative haematopoietic markers (CD34 and CD45). **B** Representative pictures of immunohistochemical analysis (*OR *Oil Red O, *SafO *safranin O and *AR *Alizarin Red from human umbilical cord estroma after 14 days with specific differentiation medium (DMEM, *AD *adipocyte medium, *CH *chondrocyte medium and *OS *osteocyte medium). **C** Histogram represents gene expression of pluripotency markers, *Nanog*,* Sox9* and *Oct4* in MSC in front of TC28a2 cell line. Real-time reverse transcriptase PCR (qRT-PCR) analysis normalized by expression of HPRT gene used as housekeeping. *P value less than 0.05 was considered statistically significant using 2ANOVA test. **D** Representative result from the NTA assay of MSC-derived EV. **E** Electron micrograph of MSC-derived EV (scale bar 100 nm). **F** Immunoblot staining for exosome markers CD63 and Calnexin as a negative control. **G** Histogram represents expression of miR-21 in MSC in front of MSC-miR21^−^ (left) and MSC-derived EV in front of MSC-miR21^−^-derived EV Real-time reverse transcriptase PCR (qRT-PCR) analysis normalized by expression of U6 gene used as housekeeping. *P value less than 0.05 was considered statistically significant using 2ANOVA test

The characterization of populations of MSC isolated from umbilical cord stroma by flow cytometry is shown in Fig. 1A. It indicated that isolated MSC contained less than 1 ± 5% of cells positive for CD45 and CD34 which are hematopoietic markers. However, these MSCs contained more than 90 ± 5% for CD105, CD90 and CD73 positive cells. These MSCs were directly differentiated towards chondrocyte, adipocyte and osteocyte lines during 14 days in culture using the adequate media (Fig. 1B). MSC were characterized by qRT-PCR measuring their pluripotent markers, *Nanog*, *Sox9* and *Oct4*, which were more significantly statistically expressed than in the TC28a2 line used as a control (Fig. 1C). NTA results revealed that the production of MSC-derived EV and their size was 160 ± 18 nm (Fig. 1D). MSC-derived EVs were visualized by electron microscopy as small vesicles, typically 40–80 nm in diameter (Fig. 1E). Western analysis of EVs revealed the level of CD63 (an exosome membrane marker protein) and the absence of calnexin (a contaminant from endoplasmic reticulum) in EV (Fig. 1F). The expression of miR-21 in transfected MSC and their derived EV was checked by qRT-PCR through Taqman probes, indicating that miR-21 was under-expressed in MSC transfected with the miR-21 inhibitor compared to MSC-mimic (Fig. 1G).

### Animal model of OA

**Fig. 2:**
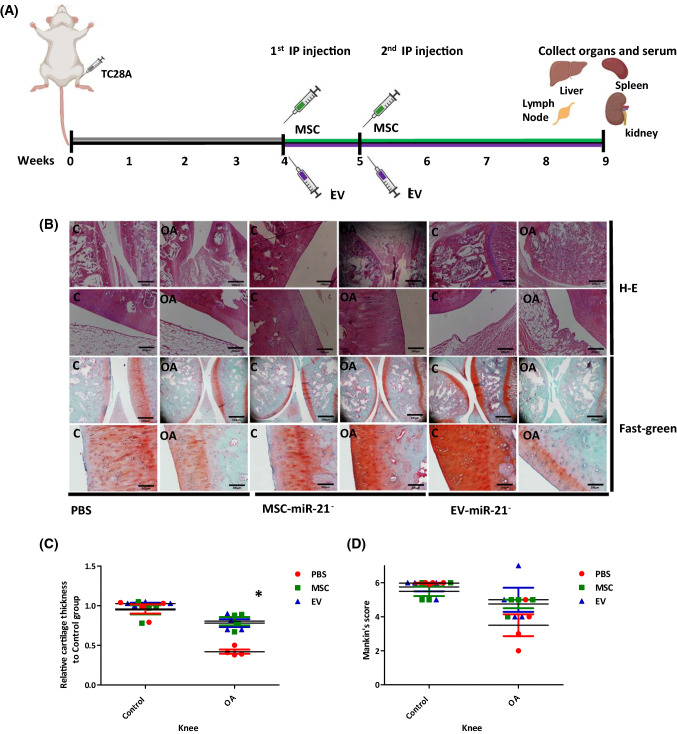
MSC-miR21^−^ and MSC-miR21^−^-derived EV reduced the cartilage degradation in the OA model. **A** Workflow of the in vivo OA model. **B** Hematoxylin–Eosin and Safranin O staining of articular cartilage obtained from OA model of Wistar male rats injected with PBS (*n* = 4) as a control group, injected with MSC-miR21^−^ (*n* = 4) or MSC-miR21^−^-derived EV treatment (*n* = 4). **C** Relative cartilage thickness and **D** Mankin’s score based on staining results. 2wayANOVA test was performed to determine the difference between treatments. *P < 0.05 compared with PBS

Cartilage degeneration induced by intraarticular injection of chondrocytes (Fig. 2A) was assessed using Safranin O/Fast green staining. Samples were cut through the medial knee joints and 5-μm sections (*n* = 4) were used. Sections were stained with Safranin O visualized under light microscope (Fig. 2B). Proteoglycan depletion in cartilage and subchondral plate thickness was assessed in accordance with the Mankin’s scoring methods (Fig. 2C, D). The rats in the MSC-miR-21^−^ (0.805 ± 0.05) and EV-miR-21^−^ (0.780 ± 0.05) treatment groups showed the amelioration of OA severity as indicated by increased safranin staining in the affected arm in front of PBS treatment 0.420 ± 0.02) (*P < 0.05) (Fig. 2C). In addition, Mankin’s score was significantly higher in OA arm compared with control one (5.97 ± 0.02 and 3.02 ± 0.04 respectively, and this rise was reduced significantly with MSC-miR-21^−^ (4.267 ± 0.02) and EV-miR-21^−^ (3.54 ± 0.04) treatments (Fig. 2D).

### Effect of treatment with MSCvs EV in serum of an OA model

**Fig. 3:**
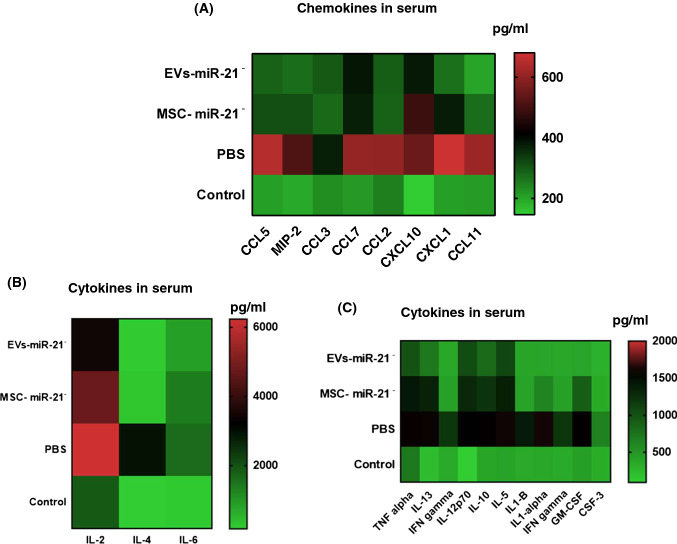
MSC-miR21^−^ and MSC-miR21^−^-derived EV reduced the pro-inflamatory markers in serum in OA model. **A** Heatmap of normalized expression levels of CCL5, MIP-2, CCL3, CCL7, CCL2, CXCL10, CXCL1 and CCL11 chemokines in serum from three experimental groups (4 animals per group) and one control group (4 animals not treated). **B** Heatmap of normalized expression levels of IL-2, IL-4 and IL-6 cytokines in serum from three experimental groups (4 animals per group) and one control group (4 animals). **C** Heatmap of normalized expression levels from TNF alpha, IL-13, IFN gamma, IL-12p70, IL-10, IL-5 cytokines in serum from three experimental groups (4 animals per group) and one control group (4 animals). Luminex multi-factor detection by xMAP technology was done. The green color in the heatmap denotes low concentration and the red color in the heatmap denotes high concentration of target proteins. EV-miR21^−^ = MSC-miR21^−^-derived EV

Luminex Immunoassay revealed that chemokines studied were statistically significant (*P < 0.05) increased in OA group treated with PBS in front of serum levels of control animal (CCL5: 601.4 ± 1.07 and 205.1 ± 1.13; MIP-2: 513.2 ± 4.13 and 206.5 ± 14.5; CCL3: 379.8 ± 12.4 and 229.8 ± 3.7; CCL7: 597.20 ± 33.22 and 213.74 ± 5.33; CCL2: 590.22 ± 56.38 and 273.84 ± 39.91; CXCL10: 556.74 ± 12.00 and 146.94 ± 4.56; CXCL1: 663.41 ± 30.93 and 204.04 ± 1.68; CCL11:614.42 ± 12.64 and 208.79 ± 5.90, respectively). After MSC-miR-21^−^ and MSC-miR-21^−^-derived EV treatments, the chemokine serum levels decreased in a statistically significant way (*P < 0.05) (CCL5: 309.30 ± 0.5 and 284.35 ± 1.07; MIP-2: 310.04 ± 8.65 and 269.93 ± 5.19; CCL3: 286.89 ± 20.09 and 302.65 ± 18.4; CCL7: 374.16 ± 47.32 and 384.81 ± 20.64; CCL2: 281.70 ± 7.80 and 301.61 ± 38.83; CXCL10: 462.77 ± 31.60 and 399.46 ± 23.62; CXCL1: 374.37 ± 19.75 and 265.18 ± 12.23; CCL11: 284.17 ± 23.68 and 202.51 ± 7.04, respectively), compared to OA group treated with PBS, these levels approaching those found in control animals (Fig. 3A). In the case of cytokines, the same differences were observed between the established groups than found in chemokines. The IL-2, IL-4 and IL-6 cytokines presented very high serum values, which is why they are represented in Fig. 3B, while the rest of the measured cytokines are presented in Fig. 3C. The cytokines studied were statistically significant (*P < 0.05) increased in OA group treated with PBS in front of serum levels of control animal (IL-2: 6340.88 ± 426.51 and 1893.93  ± 182.05; IL-4: 2900.17 ± 336.06 and 175.48 ± 0.82; IL-6: 1595.00 ± 87.17 and 223.59 ± 28.48, respectively). After MSC-miR-21^−^ and MSC-miR-21^−^-derived EV treatments, the cytokine serum levels decreased in a statistically significant way (*P < 0.05) IL-2: 4740.99 ± 155.86 and 3564.90 ± 350.77; IL-4: 270.19 ± 8.88 and 185.59 ± 6.16; IL-6: 1310.41 ± 70.62 and 820.23 ± 125.36, respectively) compared to OA group treated with PBS, these levels approaching those found in control animals (Fig. 3B).

The rest of cytokines studied were statistically significant (*P < 0.05) increased in OA group treated with PBS in front of serum levels of control animal (TNF alpha: 556.70 ± 13.44 and 254.70 ± 8.59; IL-13: 553.63 ± 13.03 and 128.17 ± 15.96; IFN gamma: 392.10 ± 4.05 and 162.84 ± 1.48; IL-12p70: 509.22 ± 17.98 and 99.62 ± 12.47; IL-10: 534.43 ± 17.58 and 171.83 ± 5.92; IL-5: 563.99 ± 49.38 and 274.87 ± 0.81, respectively). However, after MSC-miR-21^−^ and MSC-miR-21^−^-derived EV treatments, serum chemokines levels decreased although not in a statistically significant manner with respect to the OA group treated with PBS (Fig. 3C). For all the cytokines and chemokines studied, treatment with MSC-miR-21^−^-derived EV was more effective than treatment with MSC-miR-21^−^, since the values were reduced, approaching the values found in control animals.

### Effect of treatment with MSC-miR-21^−^ vs EV in organs of an OA model

**Fig. 4:**
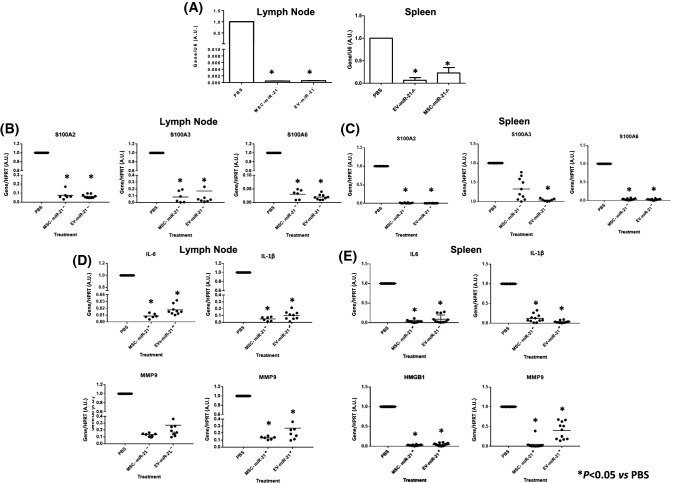
MSC-miR21^−^ and MSC-miR21^−^-derived EV reduced SASP in organs from OA model. **A** Histograms present miR-21expression normalised with U6, a housekeeping gene, in lymph node and spleen from OA model treated with PBS, MSC-miR21^−^ or MSC-miR21^−^-derived EV. **B** Histograms present SASP genes expression in lymph node from OA model treated with PBS, MSC-miR21^−^ or MSC-miR21^−^-derived EV. **C** Histograms present SASP genes expression in spleen from OA model treated with PBS, MSC-miR21^−^ or MSC-miR21^−^-derived EV. **D** Histograms present inflammaging genes expression in lymph node from OA model treated with PBS, MSC-miR21^−^ or MSC-miR21^−^-derived EV. **E** Histograms present inflammaging gene expression in lymph node from OA model treated with PBS, MSC-miR21^−^ or MSC-miR21^−^-derived EV. All genes were normalised with HPRT as a housekeeping gene. EV-miR21^−^ = MSC-miR21^−^-derived EV. *P < 0.05 compared with PBS was considered statistically significant using 2wayANOVA test

The expression of miR-21 in the lymph node and spleen from the animal model of OA was verified by qRT-PCR with Taq-man probes (Fig. 4A). It was observed that miR-21 was statistically significantly inhibited in the lymph node and spleen in the animals subjected to treatment compared to those that only were treated with PBS. Expression of *S100A2*,* S100A3*,* S100A6* genes (Fig. 4B, C) as well as interleukines (*IL-6*,* IL1-β*) and inflammation markers (*HGMB1* and *MMP9*) (Fig. 4D, E), all of them involved in SASP, were statistically significantly inhibited in those organs when the animals were treated with MSC-miR-21^−^ and MSC-miR-21^−^-derived EV *vs* OA animals treated with PBS.

### Effect of miR-21 in Inflamm-aging

**Fig. 5:**
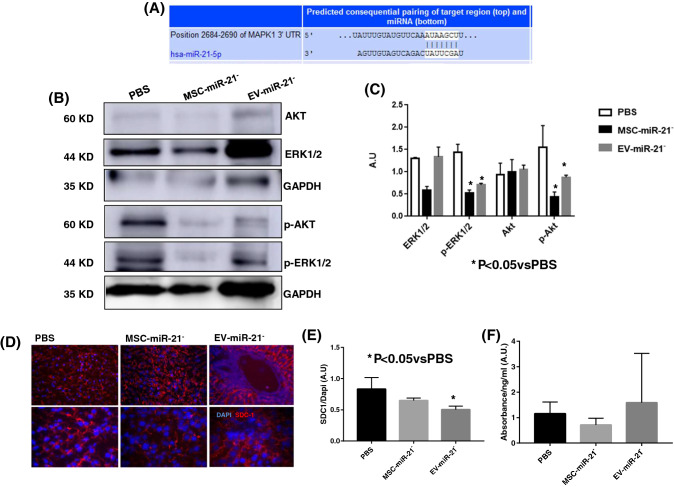
miR21 modified the MEK1/2 family. **A** In silico study using Target Scan V8.0 public repository (https://www.targetscan.org/vert_80/) revealed that MAPK family is a target of miR-21 in mammals. **B** Western blot analysis of ERK1/2, AKT, phoshor-ERK1/2, phosphor-AKT and GAPDH was used as housekeeping. **C** Plot showing their densitometry analysis normalized with respect to GAPDH. The gels were run under the same experimental conditions. **D** Representative Immunofluorescence analysis of SDC1 in liver form OA model. The photos above are at × 10 magnification and the photos below are at × 20. **E** Plot showing their densitometry analysis normalized with respect to DAPI. **F** ELISA analysis of shed SDC1 in serum from AO model. EV-miR21^−^ = MSC-miR21^−^-derived EV. *P < 0.05 compared with PBS was considered statistically significant

Studies carried out in silico using the Target Scan V8.0 public repository (https://www.targetscan.org/vert_80/), to search for miR-21 targets in mammals, revealed that the MAPK family was one of them (Fig. 5A). Therefore, we performed western blot analysis on the liver of OA model animals to find out its mechanism of action focusing on ERK1/2 and AKT. It was observed that phosphor-ERK1/2 and phosphor-AKT levels were significantly higher in PBS group in front of MSC-miR-21^−^ and MSC-miR-21^−^-derived EV treated groups (Fig. 5B, C). Syndecan-1 is involved in the communication between SASP and inflammation as well as EV production [12] which make it a good candidate to generate a new anti-inflammatory therapy. Endodomain-SDC1 was checked in the liver of the OA model by immunofluorescence (Fig. 5D) and it was observed that SDC1 levels were significantly higher in PBS group in front of MSC-miR-21^−^-derived EV treated groups (Fig. 5D, E). However, shed SDC1 was checked by ELISA (Fig. 5F) and there were no differences between groups, confirming that it was the endodomain of the protein that was affected and not its ectodomain. The treatment with EV-miR-21^−^ in the proposed rat OA model decreased the expression of endodomain of SDC-1.

## Discussion

EV carry out many different functions in organisms that include repair of tissue injuries, regulation of immune response, and inhibition of inflammation. Due to their ability to repair damaged tissues, MSC-derived EV have been widely studied in regenerative medicine. The improvement in tissue homeostasis caused by MSCs can be produced by cell-to-cell direct interaction and also by secretion of soluble factors. EV are a kind of soluble biological mediator isolated from MSCs culture media in vitro. MSC-derived EV are generated under both pathological and physiological conditions. They are primary mediators of intercellular communications by transferring mRNAs, lipids, siRNA, proteins, miRNAs, and ribosomal RNAs to adjacent or remote cells [13]. After characterization of MSC and their derived EV by different techniques (Fig. 1A–F). The efficacy of miR-21 inhibition in our MSC and their derived EV was validated by qRT-PCR using taqman probes as observed in Fig. 1G. Different disease models have been studied in MSC-derived EV experiments. There are a lot of evidences for MSC-derived EV as a new approach to cell-free treatment of OA and joint damage [14–16]. We determined the impact of modified MSC in front of their EV on OA model by evaluating the structural features of articular cartilage with Safranin O staining and Mankin’s score. Four weeks after intraarticular injection, knee joints of rats subjected to chondrocyte intraarticular injection-induced OA treated with PBS group exhibited OA pathology characterized by reduced Safranin O staining and low cell density (Fig. 2) versus the control ones. Rats in the MSC and EV treatment groups showed the amelioration of OA severity in the knee injured *versus* the rats group treated with PBS. It was found that cartilage thickness was notably lower in PBS group (*P < 0.05) compared with MSC-miR-21^−^ and EVmiR-21^−^ treatment which retarded the tissue degradation (*P < 0.05) (Fig. 2B, C). In addition, Mankin’s score was significantly higher in OA group (PBS) compared with controls (*P < 0.05), and this rise was reduced significantly with MSC and EV treatments (*P < 0.05) (Fig. 2D). All these results are similar to those published by Zhang et al*.* [17] indicating that our OA model could be used as a very accurate and fast OA animal model.

TC28a2 chondrocytes are damage factors in the animal knee which attract inflammatory cells to the injured region. CC-chemokine ligand 2 (CCL2) recruits monocytes, chemokine C-X-C motif ligand 1 (CXCL1), CXCL10 and CXCL11 attract T helper 1 (TH1) cells to injured región [18]. Our results match with those authors in that chemokines are elevated in the serum of OA animals as a response to the elevated level of pro-inflammatory markers and they decrease with the treatment of animals with MSC-miR-21^−^ and MSC-miR-21^−^-derived EV (Fig. 3). An increased production of pro-inflammatory mediators, due to aging, as well as the OA pathology development, include cytokines and chemokines besides matrix-degrading enzymes important in joint tissue destruction can be the result of the development of the SASP because of the aging as well as the OA pathology development [19]. The multigenic family of Ca^2+^-binding proteins of the EF-hand type known as S100 comprises 19 members that are differentially expressed in a large number of cell types [20]. Members of this protein family have been implicated in inflammation, and in protection from oxidative cell damage [21]. We observed the same in the OA model organs (Fig. 4), the pro-inflammatory cytokine IL-6 and the matrix-degrading enzymes MMP-9 were more highly expressed in OA tissues treated with PBS in front of MSC-miR-21^−^ and MSC-miR-21^−^-derived EV treatments (Fig. 4). On the other way microRNA profiling of sEVs from plasma of healthy subjects aged 40–100 years showed an inverse U-shaped age-related trend for miR-21-5p, consistent with senescence-associated biomarker profiles. Mensá et al*.* findings suggest that miR-21-5p/miR-217 carried by senescence sEVs spread pro-senescence signals, affecting DNA methylation and cell replication [22]. Brophy et al*.* published that injured tissue isolated from young subjects (< 40 years old) responded better than from older subjects secreting pro-inflammatory molecules promoting repair.

Wang et al. reported that miR-21 expression produced antiapoptotic and angiogenic effects of MSC with parallel effects on the phosphatase and tensin homolog (PTEN), a target of miR-21 and downstream AKT. Their results confirmed the superior cardioprotection of some MSC over others due to the involvement of miR-21 as a potential mediator of MSC therapy by improving cell survival through the PTEN/AKT pathway as cardiovascular cell therapy [23]. Kuang et al. published that exosomes derived from Wharton's jelly of human umbilical cord MSC are effective at inhibiting osteocyte apoptosis and at preventing rat osteonecrosis and that these beneficial effects are mediated by the miR-21-PTEN-AKT signaling pathway [24]. Prabowo et al*.* have published the clinical importance of this miRNA in glioneuronal tumors (gangliogliomas, GG) which are characterized by a prominent activation of the innate immune response [25]. As one of MAPK family members, the ERK1/2 is an important messenger for extracellular and intracellular signals, serving a vital role in numerous processes, including proliferation, differentiation and cellular senescence [26, 27]. Our results also confirmed that inhibition of miR-21 was affecting the ERK1/2-AKT pathway decreasing their phosphorylation in the liver of OA animals treated with MSC-miR-21^−^- and MSC-miR-21^−^-derived EV (Fig. 5).

Studies in animal models have revealed a mechanistic role of SDC1 in the regulation of contact allergies, kidney inflammation, multiple sclerosis, inflammatory bowel disease, and inflammation-associated tumorigenesis [12]. For all these reasons and also because in a proteomic shotgun study carried out in our group (Data in publication) SDC1 came out as a modulated protein when we inhibited miR-21 in MSC and their derived-EV.

SDC1 is a proteoglycan that acts as an important co-receptor for receptor tyrosine kinases and chemokine receptors, and as an adhesion receptor for structural glycoproteins of the extracellular matrix. These functions are of particular relevance in the context of inflammation and malignant disease. We studied whether the mechanism of action of MSCs and EVs with the miR-21inhibited on the ERK1/2 family and SDC1. Following appropriate immune activation through the in vivo OA model challenged by tissue damage, we focused on the study of its mechanism of action in the liver to discern the role of MSCs and their EV associated with miR-21 in inflammaging. Zhang et al*.* concluded that SDC1 played a pivotal role in ameliorating LPS-stimulated ALI models and could serve as a potential therapeutic agent for clinical application in the future [12]. We agree with these authors regarding the therapeutic importance of SDC1 to treat diseases related to inflammation (Fig. 5D–F).

Based on all of our results, we conclude that EV treatment of MSCs previously modified to contain a miR-21 antagonist is more effective in reducing systemic inflammation in age-related diseases, such as OA, than miR-21^−^-MSCs themselves. In addition, it is MSC-miR-21^−^-derived EV could modulate the ERK1/2 family through SDC1 to exert its anti-inflammatory effect in the in vivo OA model studied.

## Data Availability

Data available on request from the authors.
